# Associations Between Screen Time, Sleep, and Executive Function in School-Aged Children and Adolescents: The Moderating Role of Digital Content and Age

**DOI:** 10.3390/jcm14248842

**Published:** 2025-12-14

**Authors:** Csongor Toth, Brigitte Osser, Laura Ioana Bondar, Roland Fazakas, Florin Mihai Marcu, Nicoleta Anamaria Pascalau, Ramona Nicoleta Suciu, Bombonica Gabriela Dogaru

**Affiliations:** 1 Doctoral School of Biomedical Sciences, University of Oradea, 410087 Oradea, Romania; toth.csongor1@student.uoradea.ro (C.T.); bondar.lauraioana@student.uoradea.ro (L.I.B.); gabriela.dogaru@umfcluj.ro (B.G.D.); 2 Faculty of Physical Education and Sport, “Aurel Vlaicu” University of Arad, 310130 Arad, Romania; 3 Department of Biology and Life Sciences, Faculty of Medicine, “Vasile Goldiș” Western University of Arad, 310025 Arad, Romania; fazakas.roland@uvvg.ro; 4 Multidisciplinary Doctoral School, “Vasile Goldiș” Western University of Arad, 310025 Arad, Romania; 5 Department of Psycho Neuroscience and Recovery, Faculty of Medicine and Pharmacy, University of Oradea, 410087 Oradea, Romania; nicoleta.pascalau@didactic.uoradea.ro (N.A.P.); ramona_suciu@uoradea.ro (R.N.S.); 6 Department of Medical Rehabilitation, “Iuliu Hațieganu” University of Medicine and Pharmacy Cluj-Napoca, 410087 Cluj-Napoca, Romania; 7 Clinical Rehabilitation Hospital, 400347 Cluj-Napoca, Romania

**Keywords:** adolescents, attention, children, cognitive development, executive function, internet use, parental supervision, screen time, sleep, social media, working memory

## Abstract

**Background and Objectives**: Increased and unstructured digital exposure has raised growing concerns about its potential impact on children’s cognitive and behavioral development. Executive functions (EFs)—encompassing attention, working memory, inhibition, and cognitive flexibility—are particularly sensitive to environmental influences during development. Beyond its empirical aim, this study also sought to address a theoretical gap by clarifying how multiple dimensions of digital exposure (quantity, content quality, and sleep-related timing) jointly relate to EF performance, an area insufficiently integrated into current EF frameworks. This study aimed to examine the quantitative and qualitative dimensions of digital exposure in relation to sleep duration and EF performance among Romanian school-aged children and adolescents. **Materials and Methods**: A cross-sectional study was conducted among 142 students aged 5–19 years, using standardized cognitive tasks and structured parent questionnaires to assess screen time, digital content type, and sleep duration. Analyses included correlational tests, group comparisons, regression models, and moderation procedures. **Results**: Higher daily screen time was associated with poorer attention and working-memory performance and shorter nocturnal sleep. Children and adolescents who exceeded the recommended daily screen-time limits performed worse on executive-function measures than those within recommended limits. Digital content type and sleep duration each contributed uniquely to executive performance, and recreational digital content as well as younger age intensified the negative effects of screen exposure. **Conclusions**: Excessive daily screen time, especially involving passive or recreational content, is associated with poorer EF performance and shorter sleep in children. Adequate sleep and educational or interactive digital engagement may mitigate these effects. The findings underscore the importance of age-appropriate, structured, and balanced digital habits to support healthy cognitive development.

## 1. Introduction

In recent years, digital technologies have become deeply integrated into the daily lives of children and adolescents, reshaping how they learn, communicate, and spend leisure time. While moderate and educational screen time can enhance learning opportunities, creativity, and technological fluency, growing evidence suggests that increased and unregulated digital exposure may interfere with cognitive, emotional, and behavioral development [1,2,3,4]. These concerns have prompted global health authorities to recommend limiting recreational screen time to a maximum of two hours per day for school-aged children [5,6,7,8]. However, empirical data on how screen exposure specifically affects cognitive functions—particularly executive processes—remain mixed and context dependent.

Executive functions (EFs) represent a set of higher-order cognitive processes—such as attention control, working memory, inhibition, and cognitive flexibility—that enable goal-directed behavior, problem-solving, and self-regulation [9,10]. These functions are primarily mediated by the prefrontal cortex and continue to develop throughout childhood and adolescence [11,12,13,14]. Because EFs are essential for academic performance, emotional control, and social competence, identifying environmental factors that may enhance or hinder their development is of critical importance. Among these factors, digital media use has emerged as a potential modifiable influence.

Several studies have reported that increased daily screen exposure is negatively associated with attention span, working-memory capacity, and overall cognitive control in children [15,16,17]. Proposed mechanisms include cognitive overload, rapid task switching, and exposure to highly stimulating multimedia content, which may fragment attentional focus and impair sustained concentration [18,19,20,21,22]. Experimental evidence also suggests that frequent engagement with multitasking or fast-paced digital activities taxes neural systems involved in inhibitory control, potentially leading to diminished executive efficiency over time [23,24,25]. However, findings are not uniform; other research indicates that interactive, educational, or problem-solving digital activities may strengthen aspects of cognitive engagement and flexible thinking [26,27].

This apparent contradiction underscores the importance of content type and context in digital engagement. Passive exposure to recreational media (e.g., video streaming, social networking, or repetitive gaming) may yield different cognitive outcomes compared with structured, educational, or interactive content [28,29]. Consequently, several authors have argued that the quality of digital use may be a more meaningful predictor of cognitive development than total duration alone [30,31,32]. Yet few empirical studies have simultaneously considered both the quantitative (screen time) and qualitative (content type) dimensions of media behavior in relation to multiple EF domains [33,34,35].

Sleep represents another critical variable that may mediate or moderate the link between digital exposure and cognitive performance. Insufficient sleep duration or poor sleep quality—often linked to evening device use and blue-light exposure—has been consistently associated with impairments in attention, working memory, and academic outcomes [36,37,38]. Blue-light emission from screens can delay melatonin onset, disrupt circadian rhythms, and reduce total sleep time, particularly when devices are used close to bedtime [39,40]. Furthermore, prolonged media use may displace rest, leading to cumulative sleep deprivation, whereas adequate and regular sleep schedules appear to buffer the cognitive effects of high digital engagement [41,42,43].

Developmental stage is also an essential consideration. Younger children may be more vulnerable to the cognitive consequences of prolonged screen exposure due to the ongoing maturation of their prefrontal cortex and limited self-regulatory capacities [5,44]. In contrast, older adolescents often demonstrate greater resilience or adaptive compensation as executive networks become more established [45,46,47]. Examining age-related sensitivity to digital exposure therefore provides valuable insight into mechanisms of developmental risk and resilience.

Despite the growing literature, significant gaps remain. Much existing research relies on single indicators of screen time, overlooks qualitative differences in media content, or focuses on narrow age ranges. Moreover, cross-cultural data are scarce, and little is known about how these relationships manifest in Central or Eastern European populations, where technology access, parental monitoring, and cultural attitudes toward media may differ from those observed in Western contexts.

Therefore, from a theoretical perspective, there is a need for an integrative framework that explains how multiple dimensions of digital exposure, including quantity, content quality, and timing through sleep, interact with developmental factors to influence specific executive-function processes. Current EF models rarely incorporate digital-behavior variables or account for developmental sensitivity to media exposure, leaving this relationship conceptually underdeveloped.

Building on these gaps, the present study aimed to provide an integrative examination of the associations between daily digital exposure, sleep duration, and EF performance among Romanian school-aged children and adolescents. By combining quantitative (screen time) and qualitative (content type) indicators, the research sought to capture the multidimensional nature of media use. Additionally, it investigated whether these relationships varied by age and content type, identifying developmental and contextual factors that may influence cognitive outcomes.

Through this approach, the study contributes to clarifying the complex interplay between digital behavior, sleep, and EF. The findings are expected to inform educational policies, clinical practices, and parental guidelines aimed at fostering healthy digital engagement, adequate sleep habits, and cognitive resilience in the digital era.

## 2. Materials and Methods

### 2.1. Study Design and Setting

This research employed a cross-sectional quantitative design aimed at examining the associations between digital exposure, sleep duration, and EF performance in school-aged children and adolescents. The study was conducted at Vinga Technological High School, located in Arad County, Romania, and data collection spanned from November 2023 to May 2025.

All assessments were carried out in classroom settings during regular school hours under standardized testing conditions. Participants completed computerized and paper-based measures of EF, and parents provided complementary information regarding children’s digital media habits, sleep duration, and sociodemographic background through structured questionnaires.

### 2.2. Study Population

A total of 151 students were initially assessed for eligibility. After screening, the final sample consisted of 142 students enrolled at Vinga Technological High School, representing educational levels from early primary to late secondary grades.

Participants ranged in age from 5 to 19 years (M = 11.42, SD = 3.96), with 52% female and 48% male. For analytical purposes, participants were categorized into three developmental age groups consistent with prior literature: early childhood (5–10 years), early adolescence (11–14 years), and late adolescence (15–19 years).

Inclusion criteria required (a) regular school attendance, (b) normal or corrected-to-normal vision and hearing, (c) absence of diagnosed neurological, psychiatric, or developmental disorders, and (d) provision of written parental consent.

**Figure 1:**
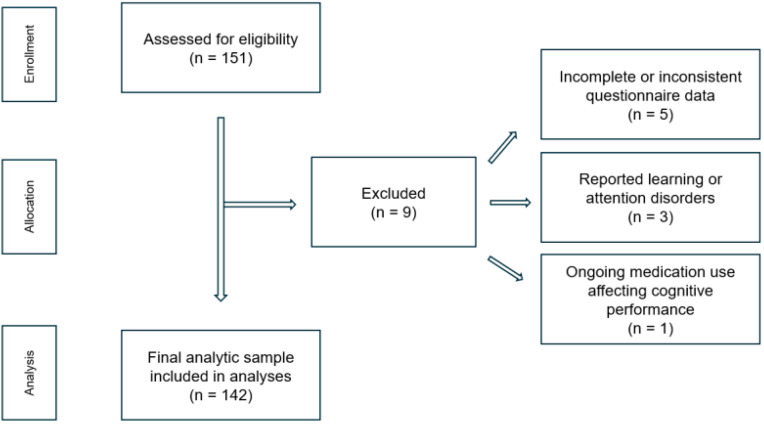
Flowchart of participant selection.

A total of nine students were excluded prior to analysis due to (a) incomplete or inconsistent questionnaire data (n = 5), (b) reported learning or attention disorders (n = 3), or (c) ongoing medication use affecting cognitive performance (n = 1). After these exclusions, the final analytic sample comprised 142 participants with complete datasets (Figure 1).

Recruitment was conducted through school announcements and information sessions with parents and teachers. Written informed consent was obtained from parents or legal guardians, and verbal assent was secured from all participating students prior to testing.

The sample size provided adequate statistical power to detect small-to-moderate effects in correlational and group-comparison analyses, consistent with previous research on digital exposure and cognitive development. A power analysis conducted using G*Power 3.1 indicated that a minimum of 118 participants would be required to detect small-to-moderate effect sizes (f^2^ = 0.10) with α = 0.05 and power (1 − β) = 0.80. The final sample (n = 142) therefore provided adequate statistical power for the study hypotheses.

### 2.3. Data Collection Instruments

Data were obtained using a combination of standardized cognitive assessments and structured questionnaires completed by both students and parents. All instruments were administered in Romanian under standardized classroom conditions by trained research staff.

- Digital Exposure Questionnaire. Parents completed a structured questionnaire adapted from prior studies on media use in school-aged children. It assessed (a) average daily screen time (in minutes per day) across weekdays and weekends, (b) type of digital content predominantly used (educational/interactive, mixed, or recreational/passive), (c) device type (smartphone, tablet, computer, television), and (d) parental monitoring practices. Average daily screen time was computed as a weighted mean of weekday and weekend exposure. Given the importance of content quality for the research aims, digital content was classified into three predefined categories based on operational definitions adapted from existing literature:
  ○ Educational/Interactive: Media designed to promote learning, problem-solving, or active engagement (e.g., educational apps, instructional videos, school-related digital tasks, interactive learning platforms).
  ○ Mixed: Cases in which children alternated between educational/interactive and recreational/passive content, with neither category exceeding approximately 60% of total digital use.
  ○ Recreational/Passive: Entertainment-oriented or passive digital activities (e.g., video streaming, social media scrolling, non-educational gaming, watching entertainment videos).

Parents first selected the predominant content category directly, and older adolescents (e.g., ages 15–19) also self-reported their predominant category when appropriate. When additional open-ended descriptions of activities or apps were provided (e.g., specific platforms or games), two independent researchers reviewed these descriptions and coded them based on the definitions above. Inter-rater agreement for this coding was high, and any discrepancies were resolved through discussion. No external classification system was used; instead, categories were adapted from prior empirical frameworks examining digital media quality in children.

- Sleep Duration and Habits. Parents reported their child’s usual bedtime and wake-up time on school days and weekends. Sleep timing was reported primarily by parents; however, older adolescents (e.g., ages 15–19) also provided self-reported sleep times when appropriate. In addition to calculating overall average nocturnal sleep duration, weekday and weekend sleep durations were analyzed separately to capture potential differences in sleep patterns and their distinct associations with digital exposure, as suggested in pediatric sleep research. A standardized sleep-quality questionnaire, such as the Pittsburgh Sleep Quality Index (PSQI), was not included due to feasibility constraints during school-based data collection and the limited age-appropriateness of the PSQI for younger children within our wide age range (5–19 years) [48]. Average nocturnal sleep duration (hours per night) was calculated as the mean of these values. Additional items evaluated sleep onset latency and perceived sleep quality. Because parents cannot reliably assess subjective sleep aspects, sleep onset latency and perceived sleep quality were answered directly by the students. This ensured that objective sleep timing variables were parent-reported, while subjective sleep experiences were self-reported by the children and adolescents.
- EF Assessment. Students completed a battery of age-appropriate, standardized cognitive tasks measuring four executive domains. Raw task scores were used in all descriptive and inferential analyses, and were extracted in their original units (accuracy %, backward span, reaction times in milliseconds). For the composite EF index, raw domain scores were standardized within age groups and averaged across the four tasks. Higher scores indicated better executive performance.
  ○ Attention was measured with a Continuous Performance Task (CPT) requiring rapid detection of target stimuli. The primary performance index was percentage accuracy (i.e., proportion of correct responses) [49,50].
  ○ Working Memory was assessed using a computerized backward-digit or spatial-span task. Performance was quantified as the maximum backward span achieved [51,52].
  ○ Inhibition was evaluated with a color–word interference (Stroop-type) task. The inhibition score corresponded to the Stroop interference effect, calculated as the reaction-time difference (in milliseconds) between incongruent and neutral trials [53,54].
  ○ Cognitive Flexibility was measured using a set-shifting task requiring alternating between rules across trials. Flexibility was indexed by the switch-cost reaction time (in milliseconds), defined as the RT difference between switch and repeat trials [55,56,57].
- Sociodemographic Form. Parents provided information on the child’s age, sex, grade level, and parental education, which were used as covariates in regression analyses.

All composite scores and questionnaire scales demonstrated satisfactory internal consistency, with Cronbach’s α values ranging between 0.78 and 0.86.

### 2.4. Procedure

Data collection was carried out during regular school hours between November 2023 and May 2025 at Vinga Technological High School, Arad County, Romania. Prior to participation, parents and students were informed about the study objectives and procedures, and written consent was obtained from parents or legal guardians.

Testing sessions were scheduled in collaboration with school staff to avoid interference with academic activities. All assessments were conducted during normal teaching days and integrated into regular school schedules; no data collection took place during school holidays. Data collection spanned both cold- and warm-season months; however, the exact proximity of testing sessions to exam periods or specific seasonal transitions was not systematically recorded.

Assessments were conducted in designated classrooms in small groups of approximately 8–12 students under standardized conditions. All executive-function tasks were administered during regular morning school hours (approximately 9:00–12:00) to ensure consistent alertness levels and to minimize fatigue or circadian influences on cognitive performance. Each session lasted about 40–50 min and included the completion of EF tasks followed by guided questionnaire administration.

Trained research staff supervised all sessions, ensuring consistent instructions and individualized assistance when needed. Parents completed their sections of the questionnaires either on-site during parent meetings or at home and returned them within one week.

All collected data were verified for completeness before entry into a secure database for subsequent analysis.

### 2.5. Ethical Considerations

The study was conducted in accordance with the ethical principles outlined in the Declaration of Helsinki and the national regulations governing research involving minors. The research protocol was reviewed and approved by the Institutional Ethics Committee of Vinga Technological High School, Arad County, Romania (approval no. 1435/2/16 October 2023).

Prior to participation, all parents or legal guardians provided written informed consent, and each student gave verbal assent after receiving an age-appropriate explanation of the study’s aims and procedures. Participation was entirely voluntary, and students could withdraw at any time without any academic or personal consequences.

All collected data were treated as confidential and anonymized using unique participant codes. Personal identifiers were removed before data analysis, and the final dataset was stored on a password-protected computer accessible only to the research team.

### 2.6. Data Analysis

Data were analyzed using IBM SPSS Statistics, Version 28.0 (IBM Corp., Armonk, NY, USA) and the PROCESS macro, Version 4.3 (available online: https://www.processmacro.org/, accessed on 14 November 2025) [58] (Hayes, 2022) for moderation analyses.

Prior to statistical testing, all variables were screened for missing data, outliers, and normality. Cases with incomplete or inconsistent data were excluded listwise, resulting in a final analytic sample of 142 participants. Normality was verified using the Shapiro–Wilk test, and homogeneity of variance was assessed with Levene’s test. Descriptive statistics (mean, standard deviation, minimum, and maximum values) were calculated for all study variables.

In addition to overall sleep patterns, weekday and weekend sleep duration were examined separately. A paired-samples *t*-test was conducted to compare weekday versus weekend sleep duration. Pearson correlations were then computed to assess the associations of weekday and weekend sleep with daily screen time and executive-function domains. These analyses allowed the identification of potential differences in how weekday and weekend sleep relate to digital exposure and EF performance.

To evaluate group differences based on digital exposure levels (≤1 h/day, 1–2 h/day, and >2 h/day), one-way analyses of variance (ANOVA) were conducted, followed by Bonferroni post hoc comparisons. Effect sizes were expressed as partial eta squared (η^2^_p_) and interpreted according to Cohen’s (1988) guidelines [59], where η^2^_p_ values of 0.01, 0.06, and 0.14 represent small, medium, and large effects, respectively.

A multiple linear regression analysis was used to determine the independent predictive value of screen time, digital content type, sleep duration, age, and maternal education on the composite EF score.

To explore conditional effects, moderation analyses were conducted using Hayes’ PROCESS Model 1, testing (a) the interaction between screen time and content type, and (b) the interaction between screen time and age group. All parametric assumptions (normality, linearity, homoscedasticity, and absence of multicollinearity) were verified prior to hypothesis testing and met the established statistical criteria. Confidence intervals (CI) 95% were computed for all primary effect estimates. For all analyses, the significance threshold was set at *p* < 0.05 (two-tailed).

### 2.7. Reliability of EF Measures

Internal consistency (Cronbach’s α) was calculated for all EF tasks to ensure adequate measurement reliability. The CPT accuracy measure, backward digit span, Stroop interference score, and switch-cost task all demonstrated acceptable to good reliability. Table 1 presents the reliability coefficients for each EF task, as well as for the digital exposure and sleep questionnaires used in the study.

### 2.8. Hypotheses of the Study

Based on previous research linking increased screen time to cognitive and behavioral outcomes in children and adolescents, the present study formulated the following hypotheses:

1. Higher levels of daily screen exposure will be negatively associated with EF performance, particularly in the domains of attention and working memory.
2. Longer sleep duration will be positively associated with EF performance, partially counteracting the negative influence of increased digital use.
3. Students exceeding the recommendation of ≤2 h/day of recreational screen time will demonstrate lower EF scores than those within the recommended limits.
4. Type of digital content will moderate the relationship between screen exposure and executive performance—with recreational or passive content (e.g., video streaming, gaming, social media) showing stronger negative associations than educational or interactive content.
5. Age will moderate the association between screen exposure and EF, such that younger children will display greater vulnerability to the negative effects of increased screen time compared to older adolescents.

## 3. Results

### 3.1. Descriptive Statistics

The descriptive analysis examined the distribution of participants according to age, sex, and level of digital exposure. The final sample comprised n = 142 students aged 5 to 19 years (M = 11.42, SD = 3.96), of whom 52% were female and 48% male. The average daily screen time was 146.7 min/day (SD = 61.4), with approximately 63% of participants exceeding the recommended limit of ≤2 h/day of recreational screen use. The mean nocturnal sleep duration was 8.1 h/night (SD = 0.9).

Regarding the qualitative dimension of digital exposure, 27% of respondents primarily engaged with educational or interactive learning materials, 36% reported mixed exposure combining learning and entertainment, and 37% mainly consumed recreational or passive content (e.g., videos, games, social media).

**Figure 2:**
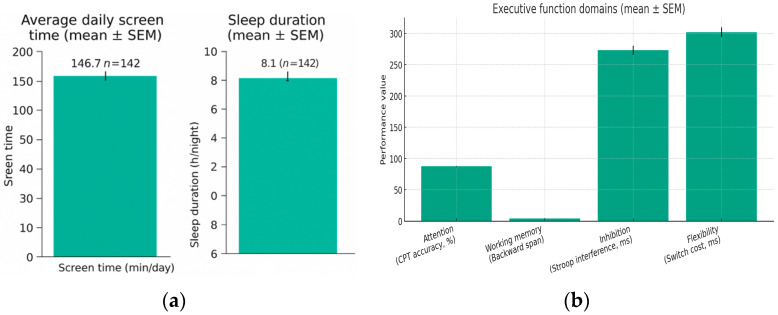
Distribution of screen time, sleep duration, and EF domains: (**a**) Average daily screen time (min/day; mean ± SEM) and average nocturnal sleep duration (h/night; mean ± SEM). (**b**) EF domains presented in original task units (mean ± SEM): CPT accuracy (%), backward span score, Stroop interference (ms), and switch-cost reaction time (ms).

Table 2 summarizes the descriptive statistics for all main variables, including the raw performance measures for each EF domain. Figure 2a presents the mean values (±SEM) for daily screen time and nocturnal sleep duration, shown as separate bar graphs. Figure 2b displays the corresponding mean values (±SEM) for EF domains using their original task metrics: CPT accuracy (%), backward span score, Stroop interference (ms), and switch-cost reaction time (ms).

Screen time showed substantial variability (35–320 min/day), whereas sleep duration remained relatively stable (6–10 h/night). EF task performance also demonstrated considerable variation across participants, reflecting developmental differences within the 5–19-year age range. This variation provided sufficient dispersion in EF scores to examine their associations with digital exposure.

All variables exhibited approximately normal distributions (*p* > 0.05, Shapiro–Wilk test), meeting the assumptions for subsequent parametric analyses.

### 3.2. Correlations Between Screen Time, Sleep, and EFs

#### 3.2.1. Associations Between Screen Time, Sleep Duration, and EF Domains

**Figure 3:**
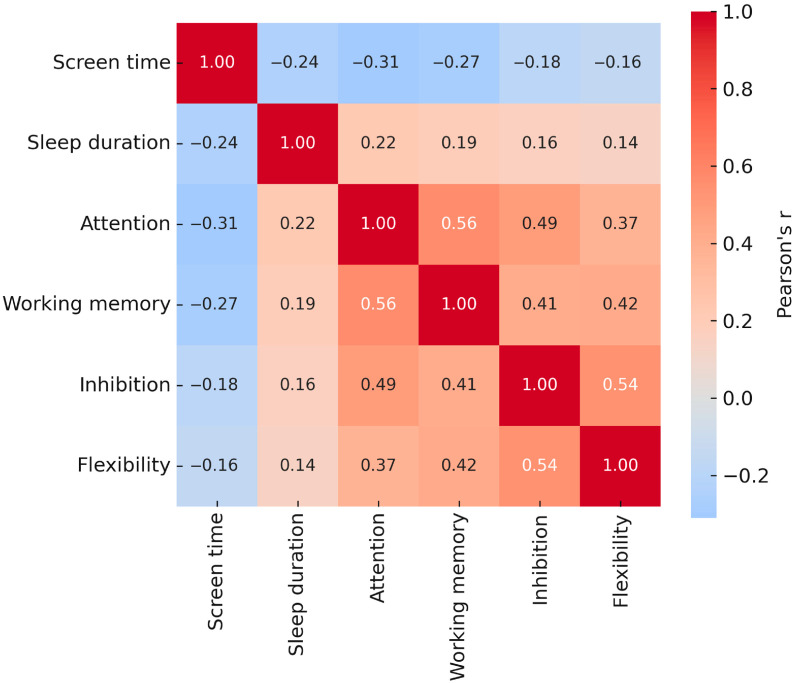
Heatmap of Pearson correlations among screen time, sleep duration, and EF domains (N = 142). Color intensity reflects the magnitude and direction of Pearson’s r coefficients (*p* < 0.05; *p* < 0.01).

Pearson correlation coefficients were computed to examine the relationships between daily screen exposure, nocturnal sleep duration, and performance across executive-function (EF) domains. As shown in Figure 3, daily screen time was negatively associated with attention accuracy and working-memory performance, and to a lesser extent with inhibition and cognitive flexibility. Screen time was also modestly associated with shorter sleep duration. These correlations indicate a consistent pattern in which greater daily exposure to digital media corresponds to reduced EF efficiency and diminished sleep opportunity.

#### 3.2.2. Associations Between Screen Time and EF Domains

**Figure 4:**
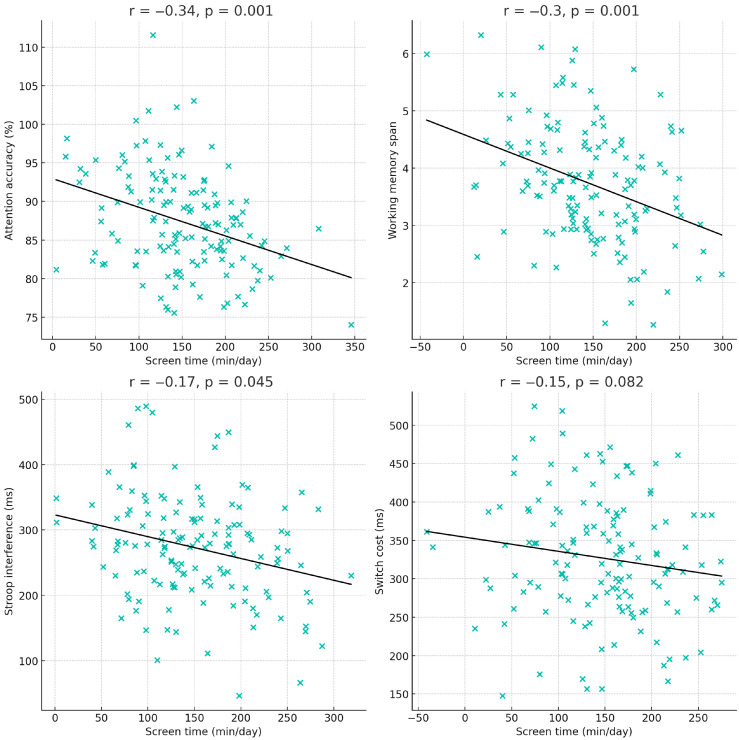
Scatterplots showing associations between daily screen time and EF domains in their original units.

To illustrate the strength and direction of these relationships using original task metrics, Figure 4 presents raw-value scatterplots for each EF domain: attention accuracy (%), backward span, Stroop interference (ms), and switch-cost reaction time (ms). Each plot includes individual data points and a regression line. The scatterplots show that higher screen exposure corresponds to lower attention accuracy and working-memory capacity, as well as higher interference and switch-cost values, reflecting less efficient inhibitory and flexible control. These visualizations provide a clearer depiction of individual variability and effect magnitude beyond the correlation matrix.

#### 3.2.3. Associations Between Screen Time and EFs by Digital Content Type

**Figure 5:**
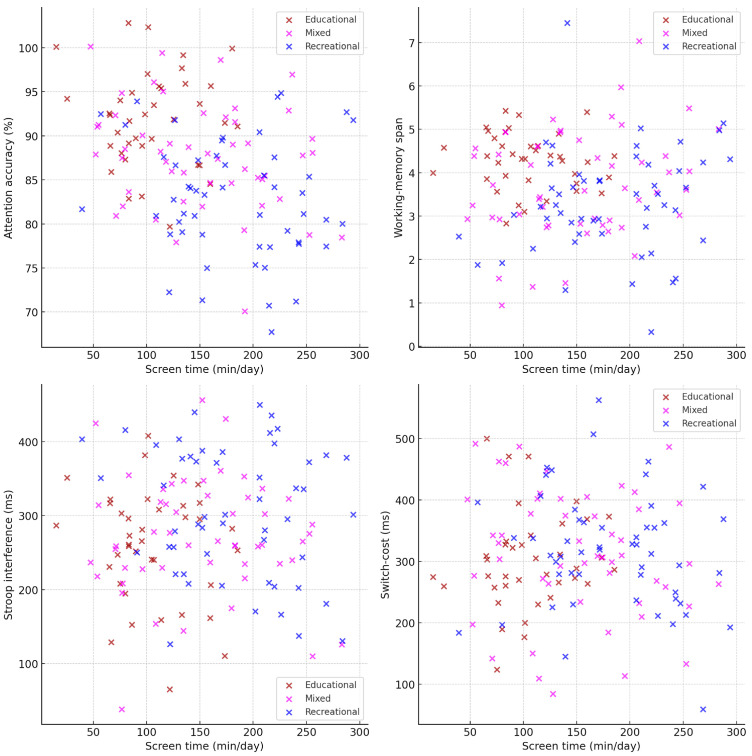
Associations between daily screen time and EF domains, separated by content type (Educational, Mixed, Recreational).

To examine whether the relationship between screen exposure and EF performance differed according to the type of digital content used, we compared participants engaging primarily with educational/interactive, mixed, or recreational/passive media. Figure 5 presents the scatterplots for all four EF domains, with the three content categories displayed using distinct colors to facilitate visual comparison.

Across EF domains, a clear pattern emerged. Children and adolescents who predominantly consumed recreational/passive content showed the steepest negative associations between daily screen time and EF performance, accompanied by greater variability in scores. Those in the mixed-content group displayed intermediate trends. In contrast, participants who primarily used educational/interactive content showed weaker and less consistent associations, suggesting that higher-quality digital engagement is less strongly related to EF reductions.

Although the three content groups are visualized within a single figure for compactness, the color-coded distributions and regression trends highlight meaningful qualitative differences. These findings indicate that the cognitive impact of screen time is moderated by content type, with recreational content amplifying the negative associations between exposure duration and EF performance.

#### 3.2.4. Screen Time and EF Performance by Digital Content Type

**Figure 6:**
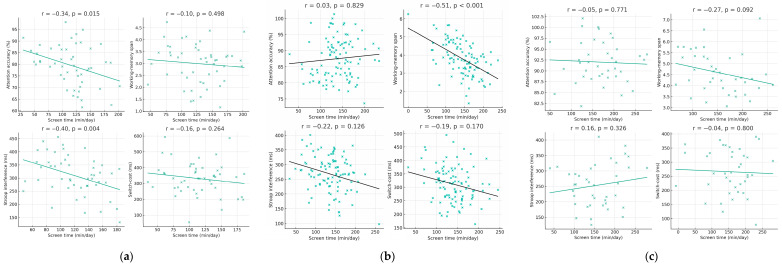
Associations between daily screen time and EF domains across age groups. Scatterplots illustrate the relationships between screen time and four EF domains (attention, working memory, inhibition, flexibility) within each developmental stage: (**a**) children aged 5–10 years, (**b**) early adolescents aged 11–14 years, and (**c**) older adolescents aged 15–19 years. Each panel includes a linear regression line, observed raw values, and corresponding Pearson correlation coefficients (r) with statistical significance (*p*-values).

Given developmental changes in executive control across childhood and adolescence, the associations between daily screen time and EF performance were examined separately for three age groups (5–10, 11–14, and 15–19 years). Figure 6 presents raw-value scatterplots for each group. The associations appear strongest during early adolescence (11–14 years), characterized by steeper regression slopes and higher correlation magnitudes, whereas effects are weaker in older adolescents (15–19 years). Children aged 5–10 years show moderate associations. These developmental patterns indicate that sensitivity to screen exposure may vary across age, potentially reflecting differences in cognitive maturation and susceptibility to cognitive load.

From a developmental perspective, these findings support the hypothesis that increased screen time may primarily interfere with attentional regulation and active working-memory maintenance—functions known to be especially vulnerable to distraction and cognitive load during middle childhood.

### 3.3. Weekday vs. Weekend Sleep Patterns

Weekday and weekend sleep duration were examined separately. As shown in Table 3, participants slept significantly fewer hours on weekdays (M = 7.9 h, SD = 0.8) compared with weekends (M = 8.5 h, SD = 0.9). A paired-samples *t*-test confirmed this difference, with t(141) = −8.12, *p* < 0.001, indicating a typical compensatory “weekend catch-up sleep” pattern.

In addition, separate correlational analyses were conducted to examine whether weekday and weekend sleep show different relationships with digital exposure and EF performance. As shown in Table 4, weekday sleep exhibited a stronger negative association with screen time and a stronger positive association with EF compared with weekend sleep.

### 3.4. Group Differences by Level of Digital Exposure

To evaluate potential threshold effects in cognitive outcomes, participants were divided into three exposure groups based on average daily screen time:

- Group 1: ≤1 h/day (n = 40);
- Group 2: 1–2 h/day (n = 52);
- Group 3: >2 h/day (n = 50).

**Figure 7:**
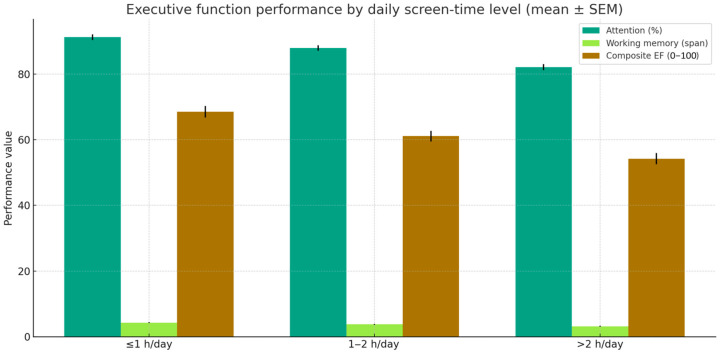
Group means (mean ± SEM) for attention accuracy (%), working memory span, and composite EF score (0–100) by daily screen-time level. Error bars denote standard error of the mean.

A one-way ANOVA was conducted to compare raw executive-function performance across these groups. Preliminary assumption checks confirmed homogeneity of variances (Levene’s test, *p* > 0.05) and normality of residuals. Significant differences emerged for attention accuracy, working memory span, and the composite EF score (Table 5, Figure 7).

Post hoc Bonferroni comparisons indicated that students in the high-exposure group (>2 h/day) performed significantly worse than those in the low-exposure group (≤1 h/day) across all three measures (*p* < 0.01), whereas differences between the intermediate (1–2 h/day) and other groups were not significant.

The effect sizes were medium to large (η^2^_p_ = 0.155–0.264), indicating substantial differences in EF performance associated with higher levels of daily screen time. This pattern suggests a nonlinear threshold effect, whereby detrimental impacts on attention, working memory, and overall executive functioning emerge primarily when screen exposure exceeds two hours per day.

Narratively, these results show that children and adolescents who consistently surpass the two-hour limit demonstrate measurable reductions in attentional accuracy, working-memory capacity, and global EF performance, whereas moderate screen use within recommended limits does not appear to impair cognitive functioning. This highlights the importance of screen-time management and suggests that both quantity and consistency of daily exposure interact with cognitive-development trajectories.

### 3.5. Multiple Regression Analysis

**Figure 8:**
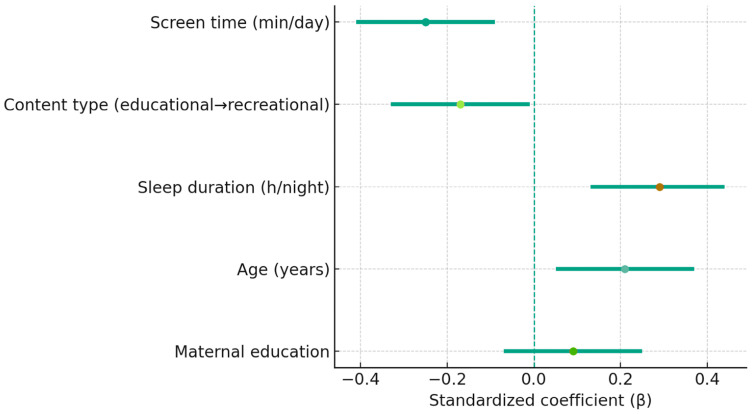
Standardized regression coefficients (β) with 95% CI for predictors of composite EF score. The vertical dashed line represents the zero reference; numerical labels include *p*-values.

A multiple linear regression analysis was conducted to assess the independent contributions of digital exposure, sleep, and demographic factors to overall executive performance. The composite EF score served as the dependent variable, and the predictors included daily screen time (minutes per day), type of digital content (coded along a continuum from educational to recreational), sleep duration (hours per night), age, and maternal education level. Standardized coefficients (β), significance values, and confidence intervals are presented in Table 6 and visualized in Figure 8.

Screen time emerged as a significant negative predictor of executive functioning (β = −0.25, *p* = 0.002), indicating that greater daily screen exposure is associated with lower EF performance even after accounting for age and sleep duration. Sleep duration represented the strongest positive predictor (β = +0.29, *p* < 0.001), underscoring the beneficial role of adequate nocturnal rest in supporting cognitive regulation. Content type also contributed significantly (β = −0.17, *p* = 0.038), revealing a gradient whereby greater engagement with recreational or passive media—relative to educational or interactive content—was associated with poorer EF outcomes. Age showed a modest positive association (β = +0.21, *p* = 0.012), while maternal education was not a significant predictor (β = +0.09, *p* = 0.265).

The overall model accounted for 21% of the variance in executive-function scores (*R*^2^ = 0.21; F(5, 136) = 7.24, *p* < 0.001), indicating a meaningful combined influence of digital habits, sleep quality, and developmental factors. Quantitatively, each additional hour of daily screen use corresponded to an estimated 0.25-SD reduction in composite EF performance, reflecting a small-to-moderate effect size with practical implications for cognitive functioning in everyday academic and social contexts.

### 3.6. Interaction Analyses

#### 3.6.1. Interaction Between Screen Time and Content Type

A moderation analysis was conducted using Hayes’ PROCESS Model 1 to examine whether the type of digital content moderated the relationship between screen time and EF performance. The interaction term between screen time and content type was statistically significant (Δ*R*^2^ = 0.04, *p* = 0.027).

Conditional effects of screen time at different levels of content type are summarized in Table 7.

Simple slope analyses revealed that, among participants who primarily consumed recreational or passive content, screen time was strongly and negatively associated with EF scores (β = −0.32, *p* = 0.004). In contrast, for participants engaging predominantly with educational or interactive content, this association was weak and non-significant (β = −0.08, *p* = 0.412).

This moderation pattern indicates that the cognitive consequences of digital exposure depend critically on content quality. Exposure to structured, goal-directed, or learning-based digital materials showed minimal association with EF outcomes, whereas passive or entertainment-oriented media use (e.g., video streaming, repetitive gaming, or unregulated social-media activity) amplified the negative relationship between exposure duration and cognitive control.

#### 3.6.2. Interaction Between Screen Time and Age

A second moderation analysis examined whether the relationship between screen time and EF performance varied across age groups. The interaction term between screen time and age was statistically significant (Δ*R*^2^ = 0.05, *p* = 0.018), indicating that age moderated the impact of digital exposure on cognitive functioning.

Simple-slope analyses showed that the negative effect of screen time was strongest among younger children (5–10 years) and became progressively weaker in the 11–14-year and 15–19-year cohorts. Graphically, this pattern corresponds to a steep descending slope for early childhood and a nearly flat slope during late adolescence, suggesting reduced sensitivity to exposure duration with increasing age. The conditional effects of screen time at each developmental level are summarized in Table 8.

From a neurodevelopmental perspective, this pattern aligns with models of prefrontal cortical maturation, in which younger children—whose executive-control systems are still developing—display heightened vulnerability to environmental overstimulation. Early excessive exposure may therefore interfere with the consolidation of self-regulatory and attentional capacities, whereas older adolescents demonstrate partial resilience due to more mature inhibitory networks.

### 3.7. Age Trends in Screen Time

**Figure 9:**
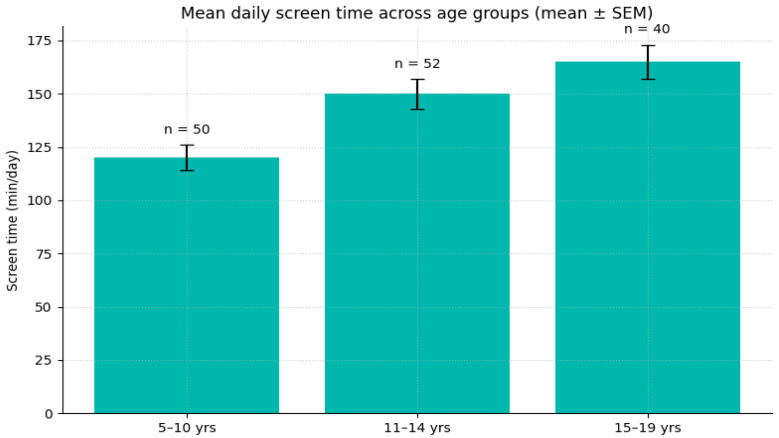
Mean daily screen time (mean ± SEM) across age groups (5–10, 11–14, and 15–19 years). Bars represent group means. Error bars denote standard error of the mean, and labels indicate group sample sizes.

To visualize developmental differences in digital exposure, the distribution of average daily screen time was examined across age groups. As shown in Figure 9, younger participants (5–10 years) reported the lowest daily screen time, whereas screen exposure increased steadily during early adolescence (11–14 years) and plateaued in older adolescents (15–19 years).

This pattern suggests that digital engagement intensifies during mid-adolescence, reflecting greater autonomy and social use of technology, while leveling off in later adolescence as usage habits stabilize.

To determine whether these observed age-group differences were statistically significant, a one-way ANOVA was conducted with age group as the between-subjects factor. The analysis showed a significant effect of age on daily screen time, F(2, 139) = 18.72, *p* < 0.001, η^2^_p_ = 0.21. Tukey post hoc comparisons indicated that children aged 5–10 years reported significantly less screen time than both early adolescents (11–14 years; *p* < 0.001) and older adolescents (15–19 years; *p* < 0.001). Early adolescents also reported slightly lower screen exposure than older adolescents (*p* = 0.041). These findings confirm that screen time increases meaningfully across developmental stages.

## 4. Discussion

The present study examined the associations between daily digital exposure, sleep duration, and EF performance among school-aged children and adolescents. The findings largely confirmed the study hypotheses, demonstrating that higher screen time was significantly associated with poorer attention and working-memory performance, shorter nocturnal sleep, and reduced overall EF efficiency. Moreover, the effects of digital exposure were moderated by both the type of digital content and the participant’s age, suggesting that qualitative and developmental factors shape the cognitive outcomes of media use.

These findings extend theoretical models of EFs by demonstrating that EF performance is differentially influenced by digital-content quality, sleep-related timing, and developmental stage. This indicates that environmental digital factors should be incorporated into contemporary EF frameworks, which traditionally do not account for the role of modern digital environments in shaping cognitive control.

These results extend previous research by providing a comprehensive analysis of both quantitative and qualitative aspects of screen exposure within a representative school-aged population. Consistent with the hypothesized model, the study highlights how prolonged and unregulated digital activity may influence cognitive control processes, particularly attention and working memory.

### 4.1. Screen Time and EF

Consistent with previous research, prolonged daily screen exposure was negatively correlated with attentional control and working-memory capacity [60,61,62]. These domains are particularly sensitive to environmental distraction and cognitive overload, as high levels of exposure to fast-paced or multitasking digital content may reduce sustained attention and limit the ability to update and manipulate information in working memory [63,64]. The small-to-moderate effect sizes observed in this study are comparable to those reported in recent meta-analyses examining the cognitive consequences of screen time in school-aged populations [65,66].

Notably, inhibitory control and cognitive flexibility were only weakly related to screen exposure, supporting the notion that not all executive domains are equally affected by digital behavior. This domain specificity aligns with neurodevelopmental evidence indicating that attentional networks mature earlier than the prefrontal systems underlying inhibition and flexibility [67,68]. Furthermore, the group-based analyses confirmed that children exceeding two hours of daily screen time performed significantly worse on attention and working-memory tasks, reinforcing the relevance of this exposure threshold for cognitive health.

Taken together, these findings suggest that the amount of daily screen time is a meaningful predictor of executive efficiency, with early-emerging attentional processes showing the greatest vulnerability to digital overstimulation.

In practical terms, the magnitude of the differences in executive functioning was meaningful. Children and adolescents who exceeded two hours of daily screen time showed approximately 9% lower attention accuracy, performed about one item lower on the working-memory span task, and demonstrated noticeably slower performance on inhibition and flexibility measures (with interference and switch-cost reaction times increased by more than 50–70 ms). These differences reflect reductions in efficiency that are detectable in everyday academic tasks requiring sustained focus, information maintenance, and rapid rule switching.

### 4.2. Sleep as a Mediating or Protective Factor

The current results revealed that shorter sleep duration was associated with both longer daily screen exposure and lower EF performance. This finding supports growing evidence that sleep plays a key linking and compensatory role between digital behavior and cognitive health [69,70,71]. Evening exposure to screens has been shown to delay sleep onset through both time displacement and the physiological effects of blue-light emission on circadian rhythms [40,72,73]. As a result, insufficient or poor-quality sleep may impair prefrontal efficiency and attentional regulation, thereby reducing overall EF [74,75,76].

The positive association between sleep duration and executive performance observed in this study suggests that adequate sleep may buffer, at least partially, the negative cognitive effects of prolonged digital exposure. This aligns with previous research demonstrating that children who maintain consistent sleep routines exhibit superior working-memory and attentional performance compared with peers who experience sleep curtailment [77,78]. Together, these findings highlight the interdependence between sleep hygiene and digital media habits, emphasizing that the cognitive consequences of screen time cannot be fully understood without considering nocturnal rest patterns

In addition, analyses separating weekday and weekend sleep duration revealed that digital exposure was more strongly associated with reduced weekday sleep, whereas weekend sleep showed weaker and less consistent associations. Weekday sleep also demonstrated a stronger relationship with EF performance, suggesting that nightly sleep obtained during structured school days may play a more influential role in supporting cognitive functioning than more variable weekend sleep. This pattern aligns with prior evidence indicating that children typically accumulate sleep deficits during weekdays and rely on weekend “catch-up sleep,” yet such compensation does not fully reverse the cognitive consequences of weekday sleep restriction [36,37,40]. Consistent sleep schedules, particularly during school days, have been shown to support attentional control and higher-order executive processes more effectively than irregular sleep patterns. These findings further underscore the importance of stable weekday sleep routines in mitigating the cognitive risks associated with higher screen engagement.

Importantly, to differentiate the effects of sleep reduction from the effects of media use on EFs, sleep duration and screen time were included as separate predictors in the regression models. This allowed us to estimate their independent contributions to EF performance. Screen time remained a significant predictor even after statistically controlling for sleep duration, indicating that media use has effects on EF that cannot be explained solely by sleep reduction. Conversely, sleep duration also showed an independent positive contribution to EF, suggesting distinct pathways through which sleep and media exposure influence cognitive functioning. Nonetheless, because the study is cross-sectional, causal direction cannot be definitively inferred, and longitudinal or experimental studies are needed to further disentangle these effects.

### 4.3. The Role of Content Type

A novel contribution of this study concerns the moderating role of digital content. The analyses revealed that screen time predicted lower executive performance primarily among participants who engaged predominantly with recreational or passive content—such as video streaming, gaming, or social media—whereas educational or interactive digital materials were not associated with cognitive detriments. This pattern supports recent evidence suggesting that the quality and purpose of digital engagement are more critical determinants of cognitive outcomes than exposure duration alone [79,80].

Interactive, goal-directed content may stimulate higher-order cognitive processes such as problem-solving, sustained attention, and working-memory updating, while passive consumption encourages minimal executive control and greater cognitive fatigue [30,81]. These findings are consistent with theoretical frameworks emphasizing that structured, feedback-based, and educational digital activities promote active learning and cognitive engagement, whereas unstructured or entertainment-based use often undermines regulatory control [82,83].

By empirically demonstrating this qualitative moderation, the present study extends prior work and highlights the importance of promoting structured, educational, and socially interactive digital experiences for children. As digital exposure continues to increase across developmental stages, understanding which types of content impose the greatest cognitive demands becomes crucial.

### 4.4. Developmental Sensitivity to Digital Exposure

The moderation analysis revealed that younger children (5–10 years) were more vulnerable to the detrimental effects of prolonged screen exposure than older adolescents. This age-dependent gradient is consistent with developmental neurocognitive models emphasizing the protracted maturation of the prefrontal cortex and self-regulatory systems [5,84,85]. During early and middle childhood, executive networks responsible for attention control, inhibition, and cognitive flexibility are still undergoing synaptic refinement and myelination, making them more sensitive to environmental overstimulation [86,87,88]. Consequently, prolonged exposure to fast-paced or unstructured digital content may interfere with the consolidation of attentional focus and working-memory strategies, reducing overall cognitive efficiency.

In contrast, older adolescents exhibited a more stable pattern of executive performance, suggesting partial adaptation or resilience as prefrontal circuits reach greater functional maturity and top-down regulation becomes more efficient [89,90,91]. This developmental pattern supports the view that the cognitive consequences of digital exposure are not uniform but vary according to age-related differences in neural plasticity and self-regulatory capacity.

These results reinforce the importance of age-tailored guidelines for screen time and parental mediation strategies. Preventive recommendations should therefore prioritize early intervention, ensuring that younger children are guided toward structured, interactive, and educational digital experiences while limiting exposure to passive recreational media.

### 4.5. Implications for Clinical Practice

The findings of this study have significant implications for both clinical and educational settings concerned with child and adolescent mental health. The observed associations between high levels of screen exposure, shorter sleep duration, and reduced EF suggest that digital behaviors should be systematically evaluated during routine pediatric and psychological assessments. Early identification of maladaptive media-use patterns may allow for targeted interventions focusing on digital hygiene, sleep regulation, and executive-skill strengthening.

From a preventive perspective, clinicians, educators, and parents should promote balanced and structured digital habits, ensuring that recreational screen time remains within the recommended limit of ≤2 h/day. Integrating media literacy programs into school curricula may help students distinguish between educational and passive digital content, encouraging cognitively enriching and goal-oriented engagement.

Given that younger children demonstrated greater vulnerability to increased screen time, early psychoeducational counseling for families is particularly important. Pediatric and school-based professionals can collaborate to implement sleep hygiene, attentional training, and digital-wellness programs, which may counteract the negative cognitive effects associated with prolonged or unregulated screen time. Such initiatives highlight the need for an interdisciplinary approach—linking healthcare, education, and family systems—to support healthy cognitive and emotional development in the digital age.

### 4.6. Research Limitations

Several limitations should be acknowledged when interpreting these results. First, the cross-sectional design precludes causal inference, as the directionality of effects between digital exposure and EF cannot be determined. Future longitudinal and experimental studies are necessary to clarify whether prolonged screen time contributes to executive deficits or whether individuals with lower self-regulatory capacity are more prone to increased digital use.

Second, data on screen time, content type, and sleep were based on parent- or self-reports, which may be influenced by recall or social-desirability biases. Sleep timing was reported primarily by parents; however, older adolescents (15–19) also provided self-reported sleep times when appropriate. The inclusion of objective measures—such as device-based tracking applications, blue-light exposure sensors, or actigraphy for sleep—would enhance the precision and ecological validity of future analyses.

Third, the study was conducted within a single educational setting in Romania, which may limit the generalizability of findings to other regions or cultural contexts. Differences in technological infrastructure, parental monitoring styles, or sociocultural norms surrounding media use could moderate these relationships.

Fourth, data were collected over an extended period (November 2023–May 2025), spanning both cold- and warm-season months; however, the exact timing of assessments relative to exam periods or specific seasonal transitions was not systematically recorded. Because stress and sleep patterns can fluctuate across seasons and during high-pressure academic weeks, some unmeasured time-related variability may have influenced the observed associations.

Finally, although the sample size provided adequate statistical power, unmeasured variables such as socioeconomic background, physical activity, and emotional well-being may also influence both digital habits and cognitive outcomes. Including these factors in future models would provide a more comprehensive understanding of the complex interplay between digital exposure and cognitive development.

Despite these limitations, the current study offers a robust empirical foundation for understanding how quantitative and qualitative aspects of digital behavior relate to children’s EF.

### 4.7. Future Research Directions

Building on these limitations and findings, future investigations should adopt longitudinal and experimental designs to clarify the temporal dynamics between digital exposure, sleep, and cognitive development. Such designs could determine whether interventions aimed at reducing screen time or enhancing sleep quality lead to measurable improvements in EF. Future studies should also incorporate validated sleep-assessment tools, such as the Pittsburgh Sleep Quality Index (PSQI), to more accurately capture sleep quality across different developmental stages.

Future work should also differentiate content-specific effects, examining the cognitive and emotional consequences of distinct media types—such as educational platforms, gaming, video streaming, or social networking—while accounting for usage context (e.g., solitary versus social interaction).

Given the age-related sensitivity identified in the present study, more refined analyses using neurodevelopmental and neuroimaging approaches are warranted to investigate how digital exposure affects prefrontal cortical maturation, attentional networks, and inhibitory control mechanisms.

Finally, interdisciplinary collaboration among psychologists, educators, neuroscientists, and technologists will be essential for developing evidence-based digital-use guidelines that balance innovation with cognitive and emotional well-being. Such collaborative efforts will contribute to personalized recommendations for healthy media engagement that account for individual, developmental, and contextual differences.

In summary, the present findings demonstrate that excessive and unstructured digital exposure—particularly to recreational content—can negatively affect attention and working memory in children. Conversely, adequate sleep duration and engagement with educational digital materials may serve as protective factors, emphasizing the need for balanced digital habits and targeted preventive strategies.

## 5. Conclusions

This study demonstrated that increased and unstructured digital exposure is associated with measurable declines in attention and working-memory performance among school-aged children and adolescents. Higher daily screen time correlated with shorter nocturnal sleep and reduced overall EF efficiency, while adequate sleep duration emerged as a significant protective factor. The negative cognitive effects of digital use were most pronounced among younger participants and those primarily engaged with recreational or passive content, indicating that both the quantity and quality of digital engagement are critical for cognitive development. The weekday–weekend sleep comparison further indicates that insufficient weekday sleep, which is more sensitive to daily digital exposure, has the strongest relevance for executive-function outcomes.

From an applied perspective, the findings support the need for balanced and developmentally appropriate digital habits, emphasizing educational and interactive content within the recommended screen-time limits (≤2 h/day). Early family-based guidance, school-level media-literacy programs, and interventions promoting healthy sleep routines may mitigate potential cognitive risks.

Overall, this research underscores the complex interplay between digital exposure, sleep, and EF, highlighting the importance of context-sensitive and age-specific strategies to foster healthy cognitive growth in the digital era.

## Figures and Tables

**Table 1:** Internal consistency (Cronbach’s α) and 95% CI of study measures.

Measure	Cronbach’s α	95% CI (Lower–Upper)
Digital Exposure Questionnaire	0.83	0.78–0.87
Sleep Duration and Habits Questionnaire	0.80	0.75–0.85
Attention (CPT)	0.81	0.76–0.85
Working Memory (Backward span Task)	0.84	0.79–0.88
Inhibition (Stroop-Type Task)	0.78	0.73–0.83
Cognitive Flexibility (Set-Shifting Task)	0.82	0.77–0.86
Composite EF Index	0.86	0.82–0.89

**Table 2:** Descriptive statistics of main variables.

Variable	M	SD	Min	Max
Screen time (min/day)	146.7	61.4	35	320
Sleep duration (hours/night)	8.1	0.9	6	10
Attention (CPT accuracy %)	87.3	6.4	68	98
Working memory (Backward span score)	3.8	1.1	1	7
Inhibition (Stroop interference, ms)	273	85	120	520
Flexibility (Switch cost, ms)	302	91	140	510
Composite EF score (scaled 0–100)	61.2	12.4	32	92

**Table 3:** Weekday and weekend sleep duration: descriptive statistics and paired-samples *t*-test.

Variable	Mean (M)	SD
Weekday sleep (h/night)	7.9	0.8
Weekend sleep (h/night)	8.5	0.9
Paired *t*-test	t(141) = −8.12	*p* < 0.001

**Table 4:** Pearson correlations of weekday and weekend sleep duration with screen time and executive-function performance.

Variable	Screen Time (Min/Day)	EF
Weekday sleep (h/night)	r = −0.24, *p* = 0.006	r = 0.29, *p* < 0.001
Weekend sleep (h/night)	r = −0.12, *p* = 0.14	r = 0.18, *p* = 0.03

**Table 5:** Mean (SD) of raw cognitive performance by exposure group.

Variable	≤1 h/Day (n = 40)	1–2 h/Day (n = 52)	>2 h/Day (n = 50)	F(2, 139)	*p*	η^2^_p_
Attention (%)	91.2 (5.8)	87.9 (6.1)	82.1 (6.7)	24.95	<0.001	0.264
Working memory (span)	4.3 (1.0)	3.8 (1.0)	3.2 (1.1)	12.71	<0.001	0.155
Composite EF (0–100)	68.5 (11.3)	61.1 (11.6)	54.2 (12.1)	16.64	<0.001	0.193

**Table 6:** Summary of multiple regression analysis predicting composite EF scores.

Predictor	β	t	*p*	95% CI
Screen time	−0.25	−3.12	0.002	−0.41, −0.09
Content type (educational → recreational)	−0.17	−2.10	0.038	−0.33, −0.01
Sleep duration	+0.29	3.66	<0.001	+0.13, +0.44
Age	+0.21	2.54	0.012	+0.05, +0.37
Maternal education	+0.09	1.12	0.265	−0.07, +0.25

Note. Model summary: *R*^2^ = 0.21; F(5, 136) = 7.24; *p* < 0.001.

**Table 7:** Conditional effects of screen time on EF performance by digital content type (Hayes PROCESS Model 1).

Content Type	β	SE	t	*p*	95% CI
Educational/Interactive	−0.08	0.09	−0.82	0.412	−0.26, +0.11
Recreational/Passive	−0.32	0.11	−2.96	0.004	−0.53, −0.10

**Table 8:** Conditional effects of screen time on EF performance at different age levels (Hayes PROCESS Model 1).

Age Group	β	SE	t	*p*	95% CI
5–10 yrs	−0.34	0.10	−3.40	0.001	−0.55, −0.14
11–14 yrs	−0.18	0.09	−1.98	0.049	−0.36, 0.00
15–19 yrs	−0.07	0.08	−0.88	0.382	−0.22, +0.08

## Data Availability

The data supporting the findings of this study are available from the corresponding author upon reasonable request. Due to privacy and ethical restrictions related to participant confidentiality, the datasets are not publicly available.

## Abbreviations

The following abbreviations are used in this manuscript:

ANOVA	Analysis of Variance
CI	Confidence Interval
EF/EFs	Executive Function/s
CPT	Continuous Performance Task
